# Initiation of Enteral Feeding with Mother’s Own Milk or Donor Human Milk in Very Preterm Infants: Impact on Bronchopulmonary Dysplasia and Other Prematurity-Related Morbidities

**DOI:** 10.3390/nu17030508

**Published:** 2025-01-30

**Authors:** Alejandro Avila-Alvarez, Sara María Fernandez-Gonzalez, Andrea Sucasas-Alonso, Alba Sanchez Ansede

**Affiliations:** 1 Neonatology Unit, Pediatrics Department, Complexo Hospitalario Universitario de A Coruña, 15006 A Coruña, Spain; andrea.sucasas.alonso@sergas.es; 2 INIBIC-Health Research Institute of A Coruña, 15006 A Coruña, Spain; 3 Gastroenterology and Hepatology Unit, Pediatrics Department, Complexo Hospitalario Universitario de A Coruña, 15006 A Coruña, Spain; sara.maria.fernandez.gonzalez@sergas.es; 4 Neonatology Unit, Lactation Support Team, Pediatrics Department, Complexo Hospitalario Universitario de A Coruña, 15006 A Coruña, Spain; alba.maria.sanchez.ansede@sergas.es

**Keywords:** mother’s own milk, donor human milk, bronchopulmonary dysplasia, prematurity, breastfeeding

## Abstract

**Background:** Bronchopulmonary dysplasia (BPD) is a major complication among preterm infants, and nutrition plays a crucial role in its prevention and management. While the nutritional superiority of human milk over preterm formula is well documented, comparisons of the protective benefits of mother’s own milk (MOM) versus donor human milk (DHM) in preterm infants are lacking. We aim to investigate if and how the use of MOM or DHM at the initiation of enteral feeding influences the development of BPD and other respiratory outcomes. **Methods:** This study evaluated the incidence of BPD and other prematurity outcomes in a cohort of 159 very preterm infants (≤32 weeks GA) who commenced enteral feeding with either MOM or DHM. **Results:** Enteral feeding was initiated with MOM in 75.5% of the infants and DHM in 24.5%. The incidence of BPD was 24.8% (39 infants), and 10.4% (16 infants) developed moderate-to-severe BPD. Univariate and multivariate analyses revealed no significant differences between the MOM and DHM groups in the rates of BPD, other respiratory outcomes, or key prematurity-related morbidities. **Conclusions:** Despite the unique bioactive properties of MOM, these findings suggest that DHM is a valid alternative that does not significantly increase the incidence of BPD or other clinical outcomes. Further studies are required to determine the relative contributions of milk volumes and feeding practices to the observed findings.

## 1. Introduction

Bronchopulmonary dysplasia (BPD) is a chronic lung disease and a significant cause of morbidity and mortality among preterm infants, particularly those born before 32 weeks of gestation [1,2]. The pathogenesis of BPD is not yet fully understood and involves a complex interplay of multiple factors, including prematurity, oxidative stress, inflammation, and disrupted alveolar development [3,4,5,6]. Despite the advances in neonatal care, the incidence of BPD has remained stable or even increased in recent years, in contrast to other prematurity-related comorbidities [6,7,8].

Although ventilator-induced damage and supplementary oxygen are among the main contributors to BPD, several other modifiable factors can influence disease incidence and severity. Nutrition is widely recognized as playing a key role in BPD prevention and management [4,5,9,10]. In fact, as neonatal intensive care has advanced, strategies to reduce BPD incidence and severity have progressively incorporated the optimization of the nutritional and feeding practices during the early postnatal period [11,12].

Mother’s own milk (MOM) is widely recognized as the gold standard for the enteral nutrition of preterm infants owing to its unique composition of bioactive components and immunological factors and its optimal nutritional profile, which collectively support growth, development, and immune protection. Studies have consistently demonstrated that MOM, compared to preterm formula (PF), is associated with reduced rates of BPD [13,14,15] and other complications of prematurity [16].

In cases where MOM is unavailable or insufficient, donor human milk (DHM) is often used as an alternative and has progressively replaced PF in neonatal units. However, many components found in MOM that protect against adverse outcomes of prematurity can be reduced, modified, or even eliminated during the processing of DHM [9,17,18] and may be related to the donor lactation stage [6,9,15,18]. Whether these changes affect clinical outcomes, including BPD, has not been extensively studied, and the published data are inconclusive [4,9,19].

Determining the differences between MOM and DHM is essential to guide feeding policies in neonatal intensive care units (NICUs) and to support interventions that promote the availability and use of MOM. We designed an observational cohort study to investigate how the use of different milk types (MOM or DHM) at the initiation of enteral feeding influences the development of BPD and other respiratory outcomes. We hypothesized that premature infants fed MOM would have lower BPD rates and improved respiratory outcomes compared to those fed DHM. As secondary objectives, we evaluated other prematurity-related outcomes and sought to identify factors associated with the initiation of feeding with DHM.

## 2. Materials and Methods

This was a retrospective cohort study of prospectively collected data carried out in a tertiary NICU of the Spanish public health system. We collected data from preterm infants ≤ 32 weeks gestational age (GA) and birth weight (BW) ≤ 1500 grams (g) who were admitted to the NICU between 1 January 2018 and 31 December 2023. Infants who died before initiation of enteral feeding or whose mothers rejected human milk were excluded from the analysis.

Information extracted from electronic medical records included demographic, perinatal, clinical, and feeding data. Primary exposure was the type of milk provided at initiation of enteral feeding (usually in the first 48 h of life according to local protocols).

Primary outcomes were the incidence of BPD, defined as the need for supplemental oxygen for at least 28 days, and moderate-to-severe BPD, defined based on respiratory support at 36 weeks postmenstrual age [11,20]. Secondary respiratory outcomes included duration of invasive mechanical ventilation (IMV), duration of non-invasive respiratory support, duration of supplemental oxygen treatment, and fraction of inspired oxygen (FiO_2_) at specific time points. Other prematurity-related outcomes, including death, were also compared between groups. Necrotizing enterocolitis (NEC) was only considered if ≥grade 2, as defined by Bell et al. [21], was considered. Patent ductus arteriosus was diagnosed by cardiac ultrasound and managed according to local protocols. Intraventricular hemorrhage was defined and graded according to Volpe [22]. All infants were screened for retinopathy of prematurity according to national guidelines [23].

The study was approved by the local Clinical Research Ethics Committee (code 2019/614) and written consent provided by all participating parents before inclusion.

### Statistical Analysis

A sample size of 159 participants was calculated based on an expected difference in proportions of 10% in main outcome (BPD), a confidence level of 90%, and a statistical power of 80%.

Descriptive data are presented as mean ± standard deviation (SD) or n (%) in the case of qualitative variables. Differences between MOM and DHM were first explored by univariate analysis. Categorical variables were analyzed using chi-squared and Fisher’s exact tests and continuous variables using the Student’s *t*-test and Mann–Whitney–Wilcoxon test. Logistic regression models were then performed for multivariate analysis of variables for which significant differences were observed in the univariate analysis. Adjusted odds ratios (ORs) and 95% confidence intervals (CIs) were calculated for selected variables. *p*-values < 0.05 were considered statistically significant. Analyses were performed using IBM SPSS statistical software v. 24.0. for Windows 

## 3. Results

**Figure 1:**
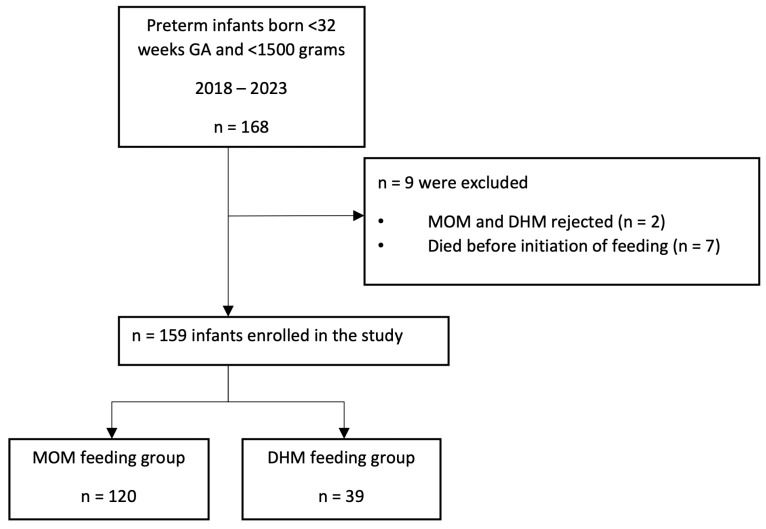
Flow chart depicting recruitment of cohort. GA, gestational age; MOM, mother’s own milk; DHM, donor human milk.

From January 2018 to December 2023, a total of 168 infants ≤ 32 weeks GA and BW ≤ 1500 g were admitted to the NICU. After applying the exclusion criteria (see flow chart, Figure 1), 159 newborns were finally included in the study. The mean GA and BW for the entire cohort were 29.4 ± 2.3 weeks and 1181.6 ± 349.7 g, respectively. Among the patients included in the study, 120 (75.5%) received MOM and 39 (24.5%) received DHM. Feeding was initiated on day 1.86 ± 0.95, with no differences between the groups. A total of thirty-nine infants (24.8%) were diagnosed with BPD: sixteen (10.4%) had moderate-to-severe BPD and nine (5.7%) died during their first hospital admission.

Significant differences were observed for certain demographic and perinatal variables between the newborns who received MOM versus DHM. As shown in Table 1, those patients who initiated feeding with DHM had a higher GA (30.2 ± 2.0 vs 29.1 ± 2.3 weeks GA; *p* = 0.013), were more often born by Caesarean section (76.9% vs 53.3%; *p* = 0.009), and were more often intubated at birth (28.2% vs 14.2%; *p* = 0.046).

As shown in Table 2, the univariate analysis revealed no significant differences between the MOM and DHM groups in terms of BPD rate, other respiratory outcomes, or prematurity-related morbidities, except for a longer duration of supplemental oxygen in the MOM group (704.2 ± 775.9 vs 306.4 ± 445.1 h; *p* = 0.007). This difference was no longer significant after adjusting by GA in the multivariate analysis (OR 0.99 [95%CI 0.9–1.0]; *p* = 0.213).

The perinatal variables that were independently associated with DHM included higher GA and being intubated in the delivery room at birth (see Table 3 for details and numerical data).

## 4. Discussion

This study, which was conducted in a consecutive cohort of 159 very preterm infants managed in the same neonatal unit, revealed no significant difference in the incidence of BPD or other outcomes associated with prematurity between the infants who received MOM versus those who received DHM upon the initiation of enteral feeding. These findings do not confirm our initial hypothesis, and they support the use of DHM as a valid alternative to MOM.

Optimal nutrition plays a crucial role in the prevention and treatment of prematurity outcomes [15,18]. In this context, MOM is the first-line option and is considered to be the gold standard for the enteral feeding of very preterm infants [4,9,18,24,25]. MOM has antioxidant properties and contains cytokines, lactoferrin, lysozymes, secretory immunoglobulin A, and growth factors that help to reduce oxidative stress and inflammation, both of which contribute to multiple prematurity outcomes, including BPD [4,6,10,13,18,26]. In fact, higher doses of MOM from birth to 36 weeks are associated with a reduced likelihood of BPD in very preterm infants [13]. MOM is therefore considered to be an inexpensive and effective strategy to reduce the risk of this costly multifactorial morbidity. In the setting of prematurity and neonatal intensive care, MOM may be unavailable or insufficient, and, in these cases, DHM is recommended as the next best alternative for preterm infants [4,9,18,24,25]. However, the composition and bioactivity of DHM may differ to those of MOM.

Differences in composition between DHM and MOM arise primarily due to the DHM processing methods, which include pasteurization, repeated freeze–thaw cycles, and the lactation stage of the milk-donating mothers [27,28]. Holder pasteurization, the most widely used technique for inactivating potentially pathogenic bacteria and viruses in human milk, involves heating the milk to 62.5 °C for 30 min. This process, however, can significantly reduce or eliminate the bioactive components that are naturally present in MOM, potentially diminishing its beneficial properties. Additionally, DHM is typically produced by pooling milk from several donors, which undergoes multiple freeze–thaw cycles. These processes can degrade or remove critical bioactive and nutritional components [29]. Lastly, most women donating milk to human milk banks are mothers of full-term, healthy infants. Since the lactation stage has a substantial influence on human milk composition, this factor also contributes to the compositional differences observed in DHM [29].

In fact, compared with MOM, DHM contains significantly lower levels of proteins, sodium, chloride, potassium, fat, and zinc, and it differs significantly in its immunologic, nutritional, and microbial properties [17]. Whether these compositional differences influence the respective benefits of each milk type in premature infants remains unclear. The majority of the studies describing the protective effects of human milk (both DHM and MOM) are based on comparisons with PF [6,8,9,10,13,15,18,26,30]. While this extensive research supports the superiority of both MOM and DHM over formula, the benefits of MOM versus DHM are less studied.

In our cohort, there were no significant differences in the incidence of BPD or other respiratory outcomes between the patients who received MOM versus DHM. These findings are in agreement with those of a recent review of three studies that examined the incidence of BPD among MOM- and DHM-fed infants and found no differences in the incidence or severity of BPD between the groups [31]. The same review reported some differences between the groups in other neonatal morbidities, infant growth, and gut microbial diversity, highlighting the unique and complex pathophysiology of BPD. A 2018 meta-analysis by Villamor-Martinez et al. also reported no differences in BPD when MOM was supplemented with DHM versus PF. However, when focusing specifically on eight observational studies, the authors found that DHM was associated with a significant reduction in the incidence of BPD (RR 0.77; 95%CI 0.62–0.96) [9]. Merino-Hernandez et al. recently published the results of an elegant study comparing the incidence of BPD among preterm infants who predominantly received MOM versus preterm DHM [19]. Despite significant differences with our study, the authors found no differences in the incidence of BPD between the MOM- and DHM-fed infants. It should be noted that, in that study, DHM was sourced from mothers of preterm infants matched by GA and days of life.

Other studies have demonstrated that earlier initiation and greater cumulative exposure to MOM, as opposed to DHM, are associated with a reduction in the incidence of moderate to severe BPD [32]. These findings align with those reported by Zhu et al. [26], who emphasized the critical role of timing in the administration of MOM in optimizing outcomes. Such studies underscore the significance of early and consistent nutritional support using MOM for preterm infants, highlighting its unique bioactive components that, according to these studies, may not be fully replicated in DHM.

Our results, however, suggest that DHM is a valid alternative to MOM and results in no significant differences in terms of neonatal outcomes. One particular benefit of DHM may be a decrease in exposure of more vulnerable infants to PF, which is known to negatively influence many prematurity-related outcomes, including NEC [33].

In our cohort, higher GA and intubation at birth were identified as variables that were independently associated with enteral feeding with DHM. The association with higher GA at birth may reflect the clinical and behavioral dynamics surrounding DHM use. Mothers of smaller, more vulnerable infants born at a lower GA may be more aware of and sensitive to the benefits of providing their own milk. Moreover, clinical teams may be more likely to provide intensive lactation counseling in this group of smaller and likely more ill infants. Consequently, mothers may prioritize efforts to provide MOM, reducing the reliance on DHM in this group, while DHM use may be more prevalent in infants with a more stable clinical course and slightly higher GA.

Regarding intubation at birth, neonates requiring intensive respiratory support, including intubation, may be less likely to receive MOM due to the combined effects of delayed lactogenesis, maternal stress, and logistical hurdles in NICU settings (especially skin-to-skin contact). In fact, separation of mother and infant and limited access to kangaroo care in intubated neonates is still common in many NICUs, and it can hinder breastfeeding and the ability to establish a robust milk supply [34].

Based on our findings, we conclude that DHM is a valid alternative for enteral nutrition in preterm infants, resulting in no significant increases in the incidence of BPD or other clinically relevant outcomes as compared with MOM. However, certain limitations of our study should be taken into account when interpreting these results. First, the single-center retrospective design of the study may limit the generalization of the results to other units with different populations or clinical practices, although this is somewhat offset by the homogeneous management of the cohort within the same neonatal unit. Second, our analysis did not include data on the volume or proportion of MOM or DHM received by the infants during hospital admission. Finally, most of the infants in the DHM group likely received at least some MOM, potentially masking the differences between the DHM and MOM groups.

## 5. Conclusions

In conclusion, our study found no differences in the incidence of BPD and other comorbidities in preterm infants <32 weeks GA who received either DHM or MOM upon commencing enteral feeding. Intubation at birth and a higher GA at birth were independently associated with enteral feeding with DHM, probably due to more intensive lactation counseling in high-risk infants and barriers to early initiation of breastfeeding in intubated infants.

## Figures and Tables

**Table 1:** Demographic and baseline variables. Bolded values denote *p* < 0.05.

	Total n = 159	MOM n = 120	DHM n = 39	*p*-Value
**Gestational age (weeks)**	**29.44 ± 2.34**	**29.18 ± 2.38**	**30.24 ± 2.04**	**0.013**
Sex (women)	78 (49.10%)	61 (50.80%)	17 (43.60%)	0.432
Birth weight (g)	1181.65 ± 349.76	1165.67 ± 345.06	1230.82 ± 364.01	0.314
Birth weight percentile	38.26 ± 24.65	40.38 ± 24.10	31.76 ± 25.90	0.059
Birth weight < 10th percentile	25 (16.40%)	16 (14.0%)	8 (23.70%)	0.165
Mother’s age	38.61 ± 58.46	39.58 ± 67.27	35.61 ± 5.30	0.714
Maternal hypertensive disorder	35 (22.00%)	26 (21.70%)	9 (23.10%)	0.853
Chorioamnionitis	26 (16.40%)	21 (17.50%)	5 (12.80%)	0.492
Antenatal steroids	149 (93.70%)	112 (93.30%)	37 (94.90%)	0.731
**Caesarean section**	**94 (59.10%)**	**64 (53.30%)**	**30 (76.90%)**	**0.009**
In vitro fertilization	22 (13.80%)	20 (16.70%)	2 (5.10%)	0.070
**Intubation in delivery room**	**28 (17.60%)**	**17 (14.20%)**	**11 (28.20%)**	**0.046**
Epinephrin in delivery room	6 (3.80%)	3 (2.50%)	3 (7.70%)	0.139
Chest compressions in delivery room	9 (5.70%)	6 (5.00%)	3 (7.70%)	0.527
Surfactant therapy	68 (42.80%)	52 (43.30%)	16 (41.00%)	0.813
Caffeine	145 (91.20%)	110 (91.70%)	35 (89.70%)	0.713
Inotropic therapy	21 (13.30%)	16 (13.30%)	5 (13.20%)	0.978
Parenteral nutrition	143 (90.50%)	107 (89.20%)	36 (94.70%)	0.737
Day when trophic feeding commenced	1.86 ± 0.95	1.84 ± 0.87	1.92 ± 1.17	0.930
Year				0.358
2018	23 (14.50%)	18 (15.00%)	5 (12.80%)	
2019	24 (15.10%)	18 (15.00%)	6 (15.40%)	
2020	33 (20.80%)	22 (18.30%)	11 (28.20%)	
2021	45 (28.30%)	38 (31.70%)	7 (17.90%)	
2022	22 (13.80%)	14 (11.70%)	8 (20.50%)	
2023	12 (7.50%)	10 (8.30%)	2 (5.10%)	

**Table 2:** Outcome variables. Bolded values denote *p* < 0.05.

Outcome Variables	Total n = 159	MOM n = 120	DHM n = 39	*p*-Value
BPD	39/157 (24.80%)	33 (27.70%)	6 (15.80%)	0.138
BPD moderate-to-severe	16/154 (10.40%)	15 (12.80%)	1 (2.70%)	0.064
Steroids for BPD	21/158 (13.30%)	19 (15.80%)	2 (5.30%)	0.094
MV	55 (34.60%)	44 (36.70%)	11 (28.20%)	0.515
Duration of MV (hours)	187.24 ± 269.50	210.37 ± 295.57	105.38 ± 118.91	0.218
Duration of NIMV (hours)	213.47 ± 205.54	229.90 ± 212.92	162.83 ± 173.88	0.085
**Duration of supplemental oxygen (hours)**	**597.29 ± 722.64**	**704.26 ± 775.91**	**306.46 ± 445.18**	**0.007**
Oxygen at home	12/155 (7.70%)	10 (8.30%)	2 (5.70%)	0.463
FiO_2_ at day 7	23.30 ± 8.39	23.38 ± 9.17	23.05 ± 5.45	0.833
FiO_2_ at day 14	23.37 ± 7.79	23.90 ± 8.82	21.70 ± 2.13	0.135
MV at day 14	10/153 (6.50%)	8 (6.90%)	2 (5.40%)	0.548
NIMV at day 28	11/156 (7.1%)	9 (7.70%)	2 (5.10%)	0.450
NEC	10/158 (6.30%)	8 (6.70%)	2 (5.30%)	0.553
Surgical NEC	6/158 (3.80%)	5 (4.20%)	1 (2.60%)	0.555
ROP > II	6/151 (4.30%)	4 (3.70%)	2 (6.10%)	0.557
IVH > II	9 (5.80%)	8 (6.90%)	1 (2.60%)	0.288
Periventricular leukomalacia	3 (1.90%)	3 (2.50%)	0 (0.0%)	0.435
Nosocomial sepsis	43 (27.00%)	32 (26.70%)	11 (28.20%)	0.851
Spontaneous intestinal perforation	5 (3.20%)	4 (3.30%)	1 (2.60%)	0.653
Anemia requiring blood transfusion	91 (57.20%)	69 (57.50%)	22 (56.40%)	0.905
PDA	21 (13.40%)	19 (16,00%)	2 (5.30%)	0.072
Ibuprofen for PDA	14 (8.90%)	13 (10.80%)	1 (2.60%)	0.104
Surgery for PDA	7 (4.40%)	5 (4.20%)	2 (5.30%)	0.535
Days in the NICU	27.74 ± 19.97	28.54 ± 21.09	25.21 ± 16.18	0.372
Days until discharge	58.35 ± 63.53	62.12 ± 70.88	45.45 ± 21.78	0.173
Duration of parenteral nutrition (days)	12.58 ± 7.96	12.52 ± 7.86	12.73 ± 8.34	0.892
Death	9 (5.70%)	7 (5.80%)	2 (5.10%)	0.614
BPD/death at 28 days of life	50 (31.60%)	42 (35.00%)	8 (21.10%)	0.107
BPD/death at 36 weeks of life	24 (15.2%)	21 (17.50%)	3 (7.90%)	0.116

BPD, bronchopulmonary dysplasia; FiO_2_, fraction of inspired oxygen; MV, mechanical ventilation; NEC, necrotizing enterocolitis; NICU, neonatal intensive care unit; NIMV, non-invasive mechanical ventilation; ROP, retinopathy of prematurity; IVH, intraventricular hemorrhage; PDA, patent ductus arteriosus.

**Table 3:** Multivariate analysis of variables related to initiation of feeding with donor human milk. *p*-values and odds ratios and corresponding 95% confidence intervals are shown. Bolded variables indicate statistical significance.

	*p*-Value	OR	CI 95%
**Gestational Age (weeks)**	**0.037**	**1.23**	**1.01–1.50**
Born by C-section	0.118	2.15	0.82–5.64
**Intubation in the DR**	**0.044**	**2.69**	**1.02–7.05**
Birth weight percentile	0.689	0.99	0.97–1.01
In vitro fertilization	0.142	0.31	0.06–1.48

DR, delivery room; OR, odds ratio; CI, confidence interval.

## Data Availability

The data that support the findings of this study are available from the corresponding author, A.A.-A., upon reasonable request.

## Abbreviations

The following abbreviations are used in this manuscript:

BPD	Bronchopulmonary Dysplasia
MOM	Mother’s Own Milk
DHM	Donor Human Milk
GA	Gestational Age
PF	Preterm Formula
BW	Birth Weight
IMV	Invasive Mechanical Ventilation
SD	Standard Deviation
OR	Odds Ratio
CI	Confidence Interval
